# Changes in Distribution of Dry Eye Disease by the New 2016 Diagnostic Criteria from the Asia Dry Eye Society

**DOI:** 10.1038/s41598-018-19775-3

**Published:** 2018-01-30

**Authors:** Takenori Inomata, Tina Shiang, Masao Iwagami, Fumika Sakemi, Keiichi Fujimoto, Yuichi Okumura, Mizu Ohno, Akira Murakami

**Affiliations:** 1 Department of Ophthalmology, Juntendo University Faculty of Medicine, Tokyo, Japan; 2 Department of Strategic Operating Room Management and Improvement, Juntendo University Faculty of Medicine, Tokyo, Japan; 3 Orange Park Medical Center, Jacksonville, FL USA; 4 Department of Non-Communicable Disease Epidemiology, London School of Hygiene and Tropical Medicine, London, UK

## Abstract

Dry eye disease (DED) is a disorder of the tear film. Here, we delineate the changes in distribution of DED after diagnostic criteria changes from the 2006 Japanese Diagnostic Criteria to the 2016 Asia Dry Eye Society criteria. We included 250 right eyes of 250 patients and all patients completed ophthalmic assessments for DED. The 2006 criteria classified patients into definite DED, probable DED, and non-DED based on subjective symptoms, tear function, and/or vital staining. The 2016 criteria eliminated probable DED and classified patients into definite DED or non-DED based on subjective symptoms and decreased tear break-up time. We examined how probable DED patients were reclassified by the 2016 criteria. By the 2006 criteria, 38.8% (97/250) of patients had definite DED, 35.6% (89/250) had probable DED, and 25.6% (64/250) had non-DED. By the 2016 criteria, 66.8% (167/250) had definite DED and 33.2% (83/250) had non-DED. Among patients with probable DED using the 2006 criteria, 79.8% (71/89) were reclassified as definite DED and 20.2% (18/89) were reclassified as non-DED using the 2016 criteria. Our data revealed that prevalence of definite DED increased because most probable DED patients were reclassified as definite DED after changes in the diagnostic criteria.

## Introduction

Dry eye disease (DED) is a disorder of the tear film characterized by tear deficiency, excessive tear evaporation and unstable tear film, causing a variety of symptoms and/or visual impairments, potentially accompanied by ocular surface damage^1,2^. Our understanding of DED has improved dramatically over the last 20 years with advancements in research. However, due to the disease’s complexity and unclear etiology, diagnostic criteria for DED are still not completely standardized worldwide.

Previously, DED was diagnosed by the 2006 Japanese Diagnostic Criteria for DED based on three assessments: subjective symptoms, tear function using either tear film break-up time (TBUT) ≤5 seconds or Schirmer test I (≤5 mm/5 min), and vital staining^3^. The 2006 criteria diagnosed definite DED with three positive items, probable DED with two positive items and non-DED with one or no positive items. In 2016, the Asia Dry Eye Society (ADES)^4^ and the Dry Eye Society Japan implemented new diagnostic criteria for DED that enabled diagnosis with only two positive items, namely subjective symptoms and decreased TBUT (≤5 seconds)^2^.

According to the 2016 criteria, probable DED is redistributed to definite DED or non-DED, but no clinical studies have investigated these changes to date. DED is the most common eye disease, affecting more than one billion people in the world^5^. Many people remain undiagnosed and experience a decreased quality of life (and decreased quality of vision)^6,7^. In addition, DED is increasing due to aging^8^, VDT (visual display terminals) syndrome accompanying smartphone and electronics use^9^, hormonal changes^10^, and stressful social environments^11^. Thus, it is necessary to clarify the change in the distribution of DED based on the 2016 diagnostic criteria to proactively recognize and identify appropriate interventions.

We aim to delineate the changes in the distribution of DED among patients, as defined by the 2006 criteria compared to the 2016 criteria.

## Results

### The Characteristics of the Participants

We enrolled 250 patients (250 right eyes assessed) in this study (Table 1). All subjects completed the examination and were eligible for analysis. The average age was 61 ± 14 years old and 76.8% of the patients were women. According to the 2006 criteria, age was significantly older in non-DED patients compared to definite DED patients, best-corrected visual acuity (BCVA) was significantly better in definite DED patients compared to non-DED patients. More women had definite DED (88.7%) and probable DED (78.7%) compared to non-DED (56.3%). Positive subjective symptom rates (definite DED: 100%, probable DED: 87.6%, non-DED: 7.8%), the Dry Eye-Related Quality-of-Life Score (DEQS)^12^ and corneal fluorescein staining (CFS) scores were significantly higher in definite DED and/or probable DED patients compared to non-DED patients. Conversely, TBUT and Schirmer test I were significantly lower in definite DED and/or probable DED patients compared to non-DED patients. Similar trends were observed in patients classified by the 2016 criteria. Age was significantly older in non-DED patients compared to definite DED patients, BCVA was significantly better in definite DED patients compared to non-DED patients. Positive subjective symptom rates, DEQS and CFS scores in definite DED patients were significantly higher compared to non-DED patients. TBUT in definite DED patients was significantly lower compared to non-DED patients. Schirmer test I was not significantly different between definite DED and non-DED patients.

**Table 1:** The characteristics of study patients.

Criteria	2006 Criteria				2016 Criteria			
Classification	Non-DED	Probable DED	Definite DED	p value	Non-DED	Definite DED	p value	Total
Characteristics ± SD	n = 64	n = 89	n = 97		n = 83	n = 167		N = 250
Age, y	65.0 ± 14.1	63.3 ± 13.5	57.4 ± 12.7	**0.001	66.6 ± 13.0	58.2 ± 12.3	**0.003	61.4 ± 14.3
Female, n (%)	36 (56.3)	70 (78.7)	86 (88.7)	***<0.001	52 (62.6)	140 (83.8)	**0.009	192 (76.8)
BCVA, LogMAR	0.1 ± 0.2	0.0 ± 0.2	0.0 ± 0.1	*0.03	0.1 ± 0.2	0.0 ± 0.1	**0.002	0.0 ± 0.2
IOP (mmHg)	13.7 ± 2.6	13.9 ± 3.1	13.8 ± 3.0	0.785	13.6 ± 2.7	13.8 ± 3.1	0.132	12.8 ± 2.9
Subjective symptom, yes (%)	5 (7.8)	78 (87.6)	97 (100.0)	***<0.001	13 (15.7)	167 (100.0)	***<0.001	180 (72.0)
DEQS score (95%CI)	11.5 ± 12.1 (3.0)	39.3 ± 26.0 (5.4)	35.7 ± 23.1 (4.6)	***<0.001	16.3 ± 21.0 (4.5)	36.4 ± 22.2 (3.4)	***<0.001	38.1 ± 23.8 (3.0)
TBUT, sec (95%CI)	4.9 ± 3.1 (0.8)	2.7 ± 1.9 (0.4)	1.6 ± 1.3 (0.3)	***<0.001	4.8 ± 3.1 (0.7)	1.8 ± 1.3 (0.2)	***<0.001	1.9 ± 2.4 (0.3)
CFS score (95%CI)	0.6 ± 0.8 (0.2)	1.5 ± 1.4 (0.3)	4.8 ± 1.9 (0.4)	***<0.001	1.1 ± 1.5 (0.3)	4.1 ± 2.5 (0.4)	***<0.001	4.1 ± 2.2 (0.3)
Schirmer I, mm (95%CI)	8.2 ± 6.3 (1.5)	7.2 ± 7.3 (1.5)	4.5 ± 5.5 (1.1)	*0.012	8.4 ± 6.5 (1.4)	4.5 ± 5.9 (0.9)	0.083	4.4 ± 6.6 (0.8)

*P* values were determined with the Student’s t-tests and one-way ANOVA for continuous variables and chi-square test for categorical variables. 95% CI; 95% confidence intervals, DED; dry eye disease, BCVA; best corrected visual acuity, IOP; intraocular pressure, DEQS; the Dry Eye Related Quality Score, TBUT; tear film break-up time, CFS; corneal fluorescein staining.

### The changes in distribution of DED subgroups per the 2006 and 2016 criteria

Table 2 shows the changes in the distribution of DED subgroups between the 2006 and 2016 criteria. According to the 2006 criteria, the population distribution of DED is definite DED (38.8%, 97/250), probable DED (35.6%, 89/250), and non-DED (25.6%, 64/250); and according to the 2016 criteria, the distribution is definite DED (66.8%, 167/250) and non-DED (33.2%, 83/250). The majority of definite DED patients (99.0%, 96/97) and all of the non-DED patients (100.0%, 64/64) as defined by the 2006 criteria remained in those same categories using the 2016 criteria. Of note, the majority of probable DED patients were redistributed primarily to definite DED (79.8%, 71/89) and few to non-DED (20.2%, 18/89) under the 2016 criteria. Table 3 shows the classification of the probable DED group by clinical examinations based on the 2006 criteria and demonstrates how they were classified under the 2016 criteria. In patients with probable DED per the 2006 criteria, the groups positive for subjective symptoms & TBUT positive (42.7%, 38/89) and subjective symptoms & TBUT & Schirmer test I (37.1%, 33/89) were reclassified as definite DED using the 2016 criteria. All other groups were reclassified as non-DED.

**Table 2:** Changes in distribution of patients with dry eye disease between the 2006 and 2016 criteria.

Criteria	2006 Criteria				
	Classification	Definite DED	Probable DED	Non-DED	Total
2016 Criteria	Definite DED	96 (99.0)	71 (79.8)	0 (0)	167 (66.8)
	Non-DED	1 (1.0)	18 (20.2)	64 (100)	83 (33.2)
	Total	97 (100)	89 (100)	64 (100)	250 (100)

Data are presented as the number of patients in each DED subgroup and the percentage of patients in each subgroup of the 2006 criteria that were reclassified into the definite DED and non-DED groups as defined by the 2016 criteria. DED; dry eye disease.

**Table 3:** Classification of the Probable DED group using the 2006 vs 2016 criteria.

2006 Criteria		2016 Criteria
Clinical examination	Positive item (%)	Classification
SS & CFS	3 (3.4)	Non-DED
SS & TBUT	38 (42.7)	Definite DED
SS & Schirmer	4 (4.5)	Non-DED
SS & TBUT & Schirmer	33 (37.1)	Definite DED
CFS & BUT	6 (6.7)	Non-DED
CFS & Schirmer	0 (0)	Non-DED
CFS & TBUT & Schirmer	5 (5.6)	Non-DED
Total	89 (100)	

Data are presented as the percentage of patients in the probable DED subgroups categorized by clinical examination using the 2006 criteria. SS; subjective symptoms, CFS; corneal fluorescence staining, TBUT; tear break-up time, Schirmer; Schirmer test I, DED; dry eye disease.

### Subgroup analyses by age and sex

**Figure 1:**
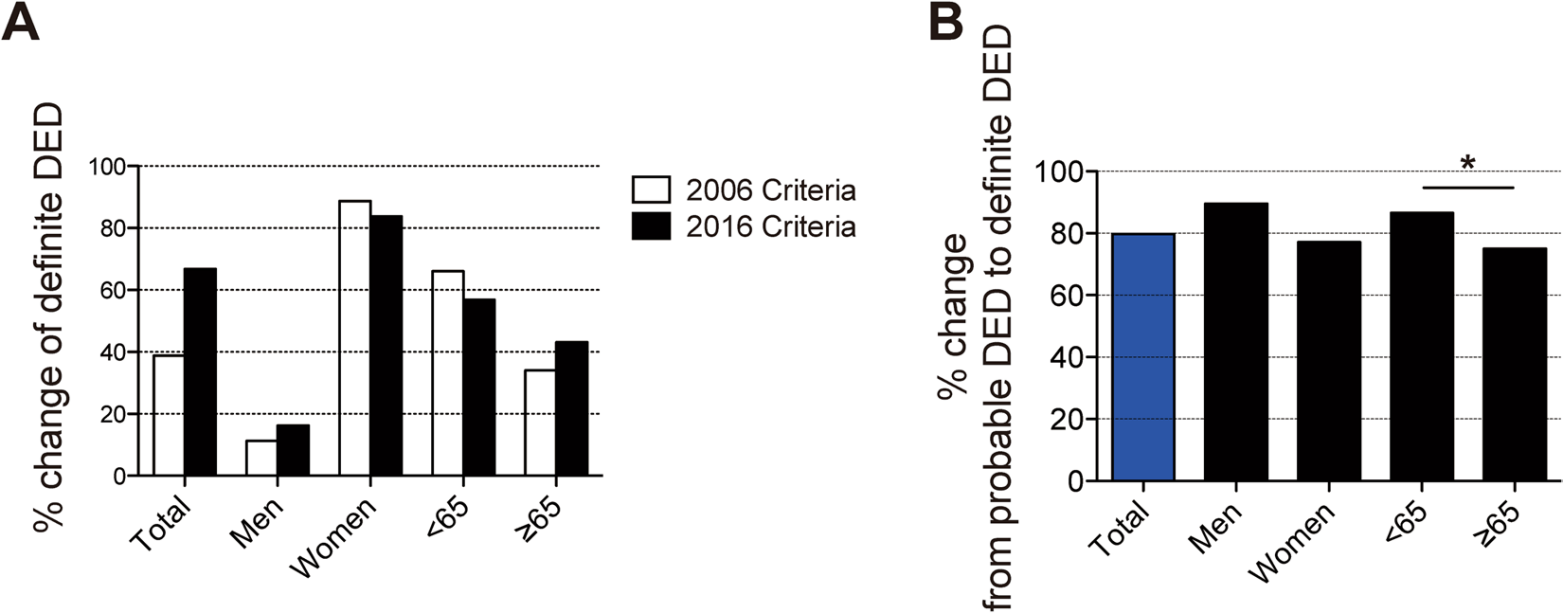
The percentage changes in patients classified as definite DED under the 2016 criteria from the 2006 criteria. (**A**) Changed distribution of definite DED between 2006 and 2016 criteria by age and sex. More men and patients ≥ 65 years old had definite DED by the 2016 criteria. (**B**) Change distribution of probable DED to definite DED between the 2006 and 2016 criteria. 79.8% of probable DED patients were reclassified as definite DED according to the 2016 criteria. In the subgroup analysis, probable DED patients who were < 65 years old were often reclassified as definite DED. Data are considered statistically significant at *p < 0.05. Chi-square test was used to compare categorical variables.

**Figure 2:**
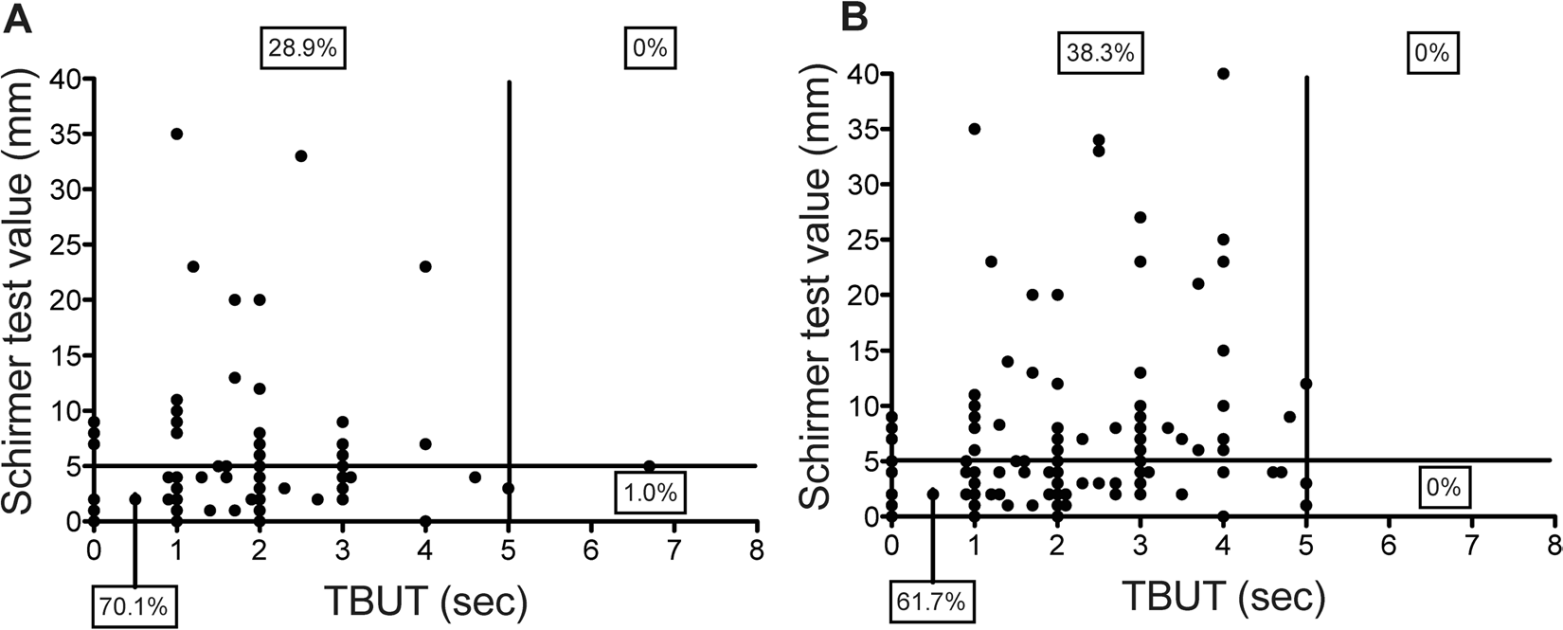
Changes in the scatter plot of Schirmer test I values and TBUT among four subtypes of patients with definite DED per the 2006 and 2016 criteria. Figures show the scatter plot of Schirmer test I results and TBUT by the (**A**) 2006 criteria and the (**B**) 2016 criteria. TBUT; tear break-up time.

Figure 1A shows a 28.0% increase in the patients classified as definite DED after the transition from the 2006 to 2016 criteria. By sex and age, the percentage changes in the patients having definite DED were 4.9% increase in men, 4.9% decrease in women, 9.1% decrease in patients <65 years old and 9.1% increase in patients ≥ 65 years old. We also examined the percentage of patients previously classified as probable DED that were reclassified as definite DED under the 2016 criteria (Fig. 2B): 79.8% in total, 89.5% in men, 77.1% in women, 86.5% in patients <65 years old and 75.0% in patients ≥ 65 years old. Patients <65 years old was primarily reclassified from probable DED to definite DED as compared to patients ≥ 65 years old (p < 0.032).

### Additional analyses

Figure 2 compares the results of the Schirmer test I and TBUT among patients with definite DED between the 2006 and 2016 criteria. In the 2006 criteria, 70.1% of definite DED patients were positive for both TBUT and the Schirmer test I, and 28.9% were positive for only TBUT (Fig. 2A). In the 2016 criteria, 61.7% of definite DED patients were positive for both TBUT and the Schirmer test I, and 38.3% were positive for only TBUT.

## Discussion

The diagnostic criteria for DED were revised by ADES and Dry Eye Society Japan in 2016^2^ and represented a standardization of DED diagnosis in Asia. With this revision, we can diagnose DED using only two items: positive subjective symptoms and decreased TBUT. Vital staining was removed from the diagnostic criteria, indicating a shift in emphasis to TBUT as a core measure of the tear film in the clinical diagnosis of DED.

From this study, we found that definite DED and non-DED patients diagnosed according to the 2006 criteria were similarly diagnosed using the 2016 criteria (Table 2). More importantly, among the probable DED patients, 79.8% were reclassified as definite DED and 20.2% were reclassified as non-DED (Table 2) under the 2016 criteria, resulting in a general increase in the population diagnosed with definite DED (Fig. 1A).

In our study, we found that most patients with probable DED, most of which were reclassified as definite DED under the 2016 criteria, were short BUT-type DED (Table 3). Short BUT-type DED is defined as short TBUT (≤5 seconds) accompanied by subjective symptoms without tear secretion deficiency or keratoconjunctival epithelium abnormality^13^. Previous studies reported that short BUT-type DED causes as much ocular discomfort and visual dysfunction as classical DED^13–16^ and requires treatment. Current studies suggest Tear Film Oriented Therapy (TFOT)^2^, focused on stabilizing the tear film layer by selectively supplementing the deficient component layer(s) of the tear film, as a possible treatment for short BUT-type DED^2^. Table 3 also shows that the new definition includes some false negatives such as patients with subjective symptoms and low Schirmer values or patients with positive CFS with either low TBUT and or Schirmer values, which may represent patients in the early stages of DED not diagnosed by the new diagnostic criteria.

Kerato-conjunctival vital staining (CFS) was excluded from the 2016 diagnostic criteria for DED because of the finding that most DED is short BUT-type DED, which occurs without any keratoconjunctival epithelium abnormality^13–16^. However, it remains an important test since the corneal epithelium is an important barrier mechanism against infection^17^ and our study revealed that 74.9% of definite DED (125/167) has positive CFS (≥3). Despite its exclusion from the 2016 diagnostic criteria, kerato-conjunctival vital staining is simple, convenient and useful in accurately assessing DED severity, which is critical for selecting appropriate interventions accordingly. Kerato-conjunctival vital staining remains our best test for assessing DED severity and definitively confirming corneal epithelial disorder and thus should continue to be performed for this purpose, even though CFS was excluded from the 2016 diagnostic criteria.

Although the Schirmer test I has been widely used in the past to evaluate tear secretion volume, it was excluded in the 2016 criteria due to the low reproducibility and sensitivity^16^. In this study, 28.9% of patients with definite DED by the 2006 criteria had normal tear volume according to the Schirmer test I (Fig. 2A). A previous study on office workers showed that 79.9% of them had normal tear volume by the Schirmer test I^16^. Here we clearly showed that most definite DED patients shared features of short BUT and tear deficiency (Fig. 2, 70.1% according to 2006, 61.7% according to 2016 criteria). A previous study showed that tear deficient DED had less subjective symptoms, but with greater clinical severity^18–20^, indicating that corneal paresthesia in DED caused the divergence between subjective symptoms and clinical signs. Unfortunately, it is difficult to detect tear deficient DED with the new diagnostic changes set forth by the 2016 criteria.

In the 2016 revision, the significance of subjective symptoms increased. Although the majority of DED patients have some correlation between subjective symptoms and clinical signs of DED, recent studies have reported cases of discordance between clinical signs of DED and subjective symptoms^21–23^. Reduction of corneal sensation in severe or chronic DED^20^, and the presence of hyperalgesia in early or mild DED^24^ can account for the discordance. Furthermore, since aging is a risk factor for dry eyes, deterioration of corneal perception due to aging^25^ causes discordance of subjective symptoms and clinical symptoms in the elderly. Therefore, the 2016 diagnostic criteria may not correctly diagnose definite DED when subjective symptoms and clinical signs are discordant.

Sex^26,27^ and age^28^ are important risk factors for DED and women are more frequently and severely affected than men^29^. However, the 2016 criteria may have revealed another at-risk population, men below 65 years old. Previously, this may have been overlooked because of the scarcity of corneal epitheliopathy in this population^29^. In our study, probable DED patients who were <65 years old were often reclassified as definite DED (Fig. 1B). Exclusion of corneal epitheliopathy from the 2016 diagnostic criteria resulting in the redistribution of DED patient classifications perhaps have made the presence of DED in men below 65 years old more apparent.

Sjogren’s syndrome also includes symptoms of dry eyes^30^. Its etiology for DED is characterized by exocrine gland inflammation, goblet cell loss and altered glycocalyx mucins, resulting in tear film instability and tear deficiency^31,32^. Currently, Sjogren’s syndrome is diagnosed using Schirmer test I and vital staining in Japan^30^. However, since the 2016 criteria removed the Schirmer test I and kerato-conjunctival vital staining from the diagnostic criteria, the emphasis of DED has shifted from tear deficiency to tear film instability, making it difficult to distinguish between these two subtypes of DED. However, in the subgroup analysis of Sjogren’s syndrome (Supplemental Table 1E), most of them had definite DED in the 2006 criteria (78.0%, 46/59) and were almost all reallocated to definite DED in the 2016 criteria (91.5%, 54/59). Our data clearly support that Sjogren’s syndrome shares features of tear film instability and tear deficiency, and the 2016 criteria included patients with DED caused by Sjogren’s syndrome even though it has excluded the Schirmer test I.

This study has a few limitations. There were a high proportion of DED patients in our study, likely due to some selection bias in choosing participants from a university hospital^5,33^. In addition, among patients with non-DED, age was significantly older and BCVA was significantly lower compared to patients with definite DED because many patients with non-DED may come for cataract examinations. This study also does not exclude patients with systemic diseases and systemic treatments. Since the examination was designed to investigate the influence of the new diagnostic criteria, rose bengal stain scores, tear osmolality and corneal sensations were not applied to all subjects. Despite these limitations, our data revealed the changes in the distribution of DED by the 2016 criteria, which eliminated the probable DED category, now a remnant of the 2006 criteria. Another limitation is our study did not assess meibomian gland dysfunction (MGD), which is due to insufficient production of meibum. However, it is fair to assume that the DED criteria has already included the MGD population because MGD shares clinical features of aqueous-deficient DED, including tear film instability and potential ocular surface compromise^34^.

In summary, we identified a changed distribution of patients in DED subgroups using the 2016 criteria. Patients previously categorized as probable DED were mostly redistributed to definite DED under the 2016 criteria. Patients categorized as definite DED and non-DED in the 2006 criteria corresponded to the respective groups in the 2016 criteria. Short BUT-type DED is not yet considered an independent disease category of DED globally, but the 2016 revision highlighted short BUT-type DED as a core concept. Specifically, tear film instability due to decreased TBUT, even without the presence of corneal epithelial damage, is an indispensable indicator in the diagnosis and treatment of DED.

We showed a change in population distribution of DED groups using the 2016 criteria. There was a significant increase in the number of patients with definite DED, primarily due to the reclassification of patients previously diagnosed as probable DED under the 2006 criteria to definite DED under the 2016 criteria. This expanded disease population emphasizes the critical importance of timely diagnosis and treatment of short BUT-type DED.

## Participants and Methods

### Study design and Participants

This cross-sectional observational study included 250 right eyes of 250 patients recruited between November 2015 and April 2017 from Juntendo University Hospital, Department of Ophthalmology, Tokyo, Japan. Written informed consent was waived due to the retrospective observational study and this study was carried out by the opt-out method of our hospital website. The clinical study was conducted under Juntendo University Hospital, Independent Ethics Committee (Approval number, 15-185) and adhered to the tenets of the Declaration of Helsinki.

### Exclusion Criteria

We excluded patients with a history of diabetes, uveitis, glaucoma, increased IOP, eye surgery, any corneal disease including herpetic keratitis, endothelial guttae and contact lens wear.

### Dry Eye Disease Diagnosis and Classification

All patients had a complete ophthalmic evaluation including measurement of BCVA, IOP, subjective symptoms using the DEQS questionnaire^12^, TBUT, CFS for kerato-conjunctival vital staining and Schirmer test I for reflex tear production. We diagnosed definite DED, probable DED and non-DED using the 2006 criteria^3^, and definite DED and non-DED using the 2016 criteria^2^. The 2006 criteria diagnosed definite DED with three positive items, probable DED with two positive items and non-DED with one or no positive items. The 2016 criteria diagnosed definite DED with two positive items, specifically positive subjective symptoms and decreased TBUT (≤5 seconds).

### Subjective Symptoms and Dry Eye-Related Quality Score (DEQS)

Subjective symptoms were collected by interviewing subjects with DED. The DEQS questionnaire was performed to assess the severity of dry eye-associated symptoms and the multifaceted effects of DED on the patients’ daily lives. The questionnaire used in this study has been evaluated for its internal validity and reliability for use in the Japanese population^12^. The score derived from this questionnaire is a subjective measurement of DED symptoms where 0 indicates the best score (no symptoms) and 100 indicates the worst score (maximal symptoms).

### **Clinical Assessments**

TBUT and kerato-conjunctival vital staining (CFS) were assessed with fluorescein sodium (Fluores Ocular Examination Test Paper, Ayumi Pharmaceutical Co., Tokyo, Japan) staining. We performed TBUT, kerato-conjunctival vital staining and subsequently Schirmer test I. To avoid the influence of TBUT and kerato-conjunctival vital staining on the Schirmer I test, we waited a minimum interval of 15 min between tests.

### Tear Break-up Time (TBUT)

TBUT is measured using a fluorescein dye according to the standard method^2^. To minimize the effect on tear volume and TBUT, a small quantity of dye was administered with a wetted fluorescein strip. After the dye was instilled, the subject was instructed to blink three times to ensure adequate mixing of the dye with the tears. The time interval between the last blink and the appearance of the first dark spot on the cornea was measured with a stopwatch. The mean value of the three measurements was used. The cutoff value of TBUT ≤5 seconds was used to diagnose DED^2^.

### Kerato-conjunctival Vital Staining (Cornea Fluorescence Staining; CFS)

CFS was graded according to the van Bijsterveld grading system^35^. Van Bijsterveld described a scoring system that divides the ocular surface into three zones: nasal bulbar conjunctiva, temporal bulbar conjunctiva, and cornea. Each zone was evaluated on a scale of 0 to 3, with 0 indicating no staining and 3 indicating confluent staining. The maximum possible score is 9.

### Schirmer test I

The Schirmer test I was performed without topical anesthesia, following all other examinations. Schirmer’s test strips (Ayumi Pharmaceutical Co., Tokyo, Japan) were placed at the outer one-third of the temporal lower conjunctival fornix for 5 minutes. The strips were then removed, and the length of dampened filter paper (in mm) was recorded.

### Statistical analyses

We examined the characteristics of the patients according to the DED classifications based on the 2006 and 2016 criteria. Student’s t test was used to compare continuous variables between two groups. One-way ANOVA with Bonferroni post hoc test was used to compare between three groups. Chi-square test was used to compare categorical variables. Data are presented as mean ± standard deviation (SD) or proportion. We analyzed the distribution changes of DED in the population between the 2006 and 2016 criteria. In addition, we calculated the proportion changes of definite DED between the 2006 and 2016 criteria.

We performed a subgroup analysis of probable DED patients classified by the 2006 criteria and examined how those subgroups were reallocated using the 2016 criteria. In addition, we conducted subgroup analysis by age, sex and Sjogren’s syndrome. To assess the relationship between TBUT and Schirmer test I, we performed scatter plot analysis of definite DED patients between the 2006 and 2016 criteria.

The data and statistical analyses were conducted using STATA version 14 (Stata Corp, Texas). A p-value of <0.05 was considered statistically significant.

## Electronic supplementary material

Supplementary information

## References

[1] Craig JP (2017). TFOS DEWS II Definition and Classification Report. Ocul Surf.

[2] Tsubota K (2017). New Perspectives on Dry Eye Definition and Diagnosis: A Consensus Report by the Asia Dry Eye Society. Ocul Surf.

[3] Shimazaki J (2007). Dry Eye Research Group. Definition and diagnosis of dry eye 2006. Atarashii Ganka (J of Eye).

[4] Horai R (2015). Microbiota-Dependent Activation of an Autoreactive T Cell Receptor Provokes Autoimmunity in an Immunologically Privileged Site. Immunity.

[5] Gayton JL (2009). Etiology, prevalence, and treatment of dry eye disease. Clin Ophthalmol.

[6] Goto E, Yagi Y, Matsumoto Y, Tsubota K (2002). Impaired functional visual acuity of dry eye patients. Am J Ophthalmol.

[7] Kaido M, Ishida R, Dogru M, Tsubota K (2011). The relation of functional visual acuity measurement methodology to tear functions and ocular surface status. Jpn J Ophthalmol.

[8] Ding J, Sullivan DA (2012). Aging and dry eye disease. Exp Gerontol.

[9] Courtin R (2016). Prevalence of dry eye disease in visual display terminal workers: a systematic review and meta-analysis. BMJ Open.

[10] Truong S, Cole N, Stapleton F, Golebiowski B (2014). Sex hormones and the dry eye. Clin Exp Optom.

[11] Yilmaz U, Gokler ME, Unsal A (2015). Dry eye disease and depression-anxiety-stress: A hospital-based case control study in Turkey. Pak J Med Sci.

[12] Sakane Y (2013). Development and validation of the Dry Eye-Related Quality-of-Life Score questionnaire. JAMA Ophthalmol.

[13] Toda I, Shimazaki J, Tsubota K (1995). Dry eye with only decreased tear break-up time is sometimes associated with allergic conjunctivitis. Ophthalmology.

[14] Kaido M, Ishida R, Dogru M, Tsubota K (2012). Visual function changes after punctal occlusion with the treatment of short BUT type of dry eye. Cornea.

[15] Koh S (2008). Effects of suppression of blinking on quality of vision in borderline cases of evaporative dry eye. Cornea.

[16] Yokoi N (2015). Importance of tear film instability in dry eye disease in office workers using visual display terminals: the Osaka study. Am J Ophthalmol.

[17] Mantelli F, Mauris J, Argueso P (2013). The ocular surface epithelial barrier and other mechanisms of mucosal protection: from allergy to infectious diseases. Curr Opin Allergy Clin Immunol.

[18] Xu KP, Yagi Y, Tsubota K (1996). Decrease in corneal sensitivity and change in tear function in dry eye. Cornea.

[19] Hay EM (1998). Weak association between subjective symptoms or and objective testing for dry eyes and dry mouth: results from a population based study. Ann Rheum Dis.

[20] Bourcier T (2005). Decreased corneal sensitivity in patients with dry eye. Invest Ophthalmol Vis Sci.

[21] Nichols KK, Nichols JJ, Mitchell GL (2004). The lack of association between signs and symptoms in patients with dry eye disease. Cornea.

[22] Begley CG (2003). The relationship between habitual patient-reported symptoms and clinical signs among patients with dry eye of varying severity. Invest Ophthalmol Vis Sci.

[23] Schein OD, Tielsch JM, Munoz B, Bandeen-Roche K, West S (1997). Relation between signs and symptoms of dry eye in the elderly. A population-based perspective. Ophthalmology.

[24] McMonnies CW (2017). The potential role of neuropathic mechanisms in dry eye syndromes. J Optom.

[25] Benitez-Del-Castillo JM (2007). Relation between corneal innervation with confocal microscopy and corneal sensitivity with noncontact esthesiometry in patients with dry eye. Invest Ophthalmol Vis Sci.

[26] Galor A (2011). Prevalence and risk factors of dry eye syndrome in a United States veterans affairs population. Am J Ophthalmol.

[27] Chia EM (2003). Prevalence and associations of dry eye syndrome in an older population: the Blue Mountains Eye Study. Clin Exp Ophthalmol.

[28] Shah S, Jani H (2015). Prevalence and associated factors of dry eye: Our experience in patients above 40 years of age at a Tertiary Care Center. Oman J Ophthalmol.

[29] Schaumberg DA (2013). Patient reported differences in dry eye disease between men and women: impact, management, and patient satisfaction. PloS one.

[30] Fujibayashi T, Sugai S, Miyasaka N, Hayashi Y, Tsubota K (2004). Revised Japanese criteria for Sjogren’s syndrome (1999): availability and validity. Mod Rheumatol.

[31] Mantelli F, Argueso P (2008). Functions of ocular surface mucins in health and disease. Curr Opin Allergy Clin Immunol.

[32] Gipson IK, Spurr-Michaud SJ, Senchyna M, Ritter R, Schaumberg D (2011). Comparison of mucin levels at the ocular surface of postmenopausal women with and without a history of dry eye. Cornea.

[33] Kawashima M (2017). A Clinic-based Survey of Clinical Characteristics and Practice Pattern of Dry Eye in Japan. Adv Ther.

[34] Schaumberg DA (2011). The international workshop on meibomian gland dysfunction: report of the subcommittee on the epidemiology of, and associated risk factors for, MGD. Invest Ophthalmol Vis Sci.

[35] van Bijsterveld OP (1969). Diagnostic tests in the Sicca syndrome. Arch Ophthalmol.

