# Author Correction: Changes in Distribution of Dry Eye Disease by the New 2016 Diagnostic Criteria from the Asia Dry Eye Society

**DOI:** 10.1038/s41598-018-28587-4

**Published:** 2018-07-04

**Authors:** Takenori Inomata, Tina Shiang, Masao Iwagami, Fumika Sakemi, Keiichi Fujimoto, Yuichi Okumura, Mizu Ohno, Akira Murakami

**Affiliations:** 1 Department of Ophthalmology, Juntendo University Faculty of Medicine, Tokyo, Japan; 2 Department of Strategic Operating Room Management and Improvement, Juntendo University Faculty of Medicine, Tokyo, Japan; 3 Orange Park Medical Center, Jacksonville, FL USA; 4 Department of Non-Communicable Disease Epidemiology, London School of Hygiene and Tropical Medicine, London, UK

Correction to: *Scientific Reports* 10.1038/s41598-018-19775-3, published online 30 January 2018

The original version of this Article contained errors.

In the Abstract

“By the 2016 criteria, 66.8% (167/250) had definite DED and 31.2% (83/250) had non-DED.”

now reads:

“By the 2016 criteria, 66.8% (167/250) had definite DED and 33.2% (83/250) had non-DED.”

In the Introduction section

“In 2016, the Asia Cornea Society (ADES)^4^ and the Dry Eye Society Japan implemented new diagnostic criteria for DED that enabled diagnosis with only two positive items, namely subjective symptoms and decreased TBUT ( ≤ 5 seconds)^2^”

now reads:

“In 2016, the Asia Dry Eye Society (ADES)^4^ and the Dry Eye Society Japan implemented new diagnostic criteria for DED that enabled diagnosis with only two positive items, namely subjective symptoms and decreased TBUT ( ≤ 5 seconds)^2^”

In Table 2, the incorrect value for ‘Non-DED total’ was given,

“78 (31.2)”

now reads:

“83 (33.2)”

As a result in the Results section,

“Table 2 shows the changes in the distribution of DED subgroups between the 2006 and 2016 criteria. According to the 2006 criteria, the population distribution of DED is definite DED (38.8%, 97/250), probable DED (35.6%, 89/250), and non-DED (25.6%, 64/250); and according to the 2016 criteria, the distribution is definite DED (66.8%, 167/250) and non-DED (31.2%, 78/250).”

now reads:

“Table 2 shows the changes in the distribution of DED subgroups between the 2006 and 2016 criteria. According to the 2006 criteria, the population distribution of DED is definite DED (38.8%, 97/250), probable DED (35.6%, 89/250), and non-DED (25.6%, 64/250); and according to the 2016 criteria, the distribution is definite DED (66.8%, 167/250) and non-DED (33.2%, 83/250).”

Additionally in the Results section,

“Figure 1 A shows a 33.0% increase in the patients classified as definite DED after the transition from the 2006 to 2016 criteria.”

now reads:

“Figure 1 A shows a 28.0% increase in the patients classified as definite DED after the transition from the 2006 to 2016 criteria.”

Furthermore, in the legend of Figure 1,

“(A) Changed distribution of definite DED between 2006 and 2016 criteria by age and sex. More men and patients > 65 years old had definite DED by the 2016 criteria.”

now reads:

“(A) Changed distribution of definite DED between 2006 and 2016 criteria by age and sex. More men and patients ≥ 65 years old had definite DED by the 2016 criteria.”

As a result in the Results section,

“By sex and age, the percentage changes in the patients having definite DED were 4.9% increase in men, 4.9% decrease in women, 9.1% decrease in patients < 65 years old and 9.1% increase in patients > 65 years old.”

now reads:

“By sex and age, the percentage changes in the patients having definite DED were 4.9% increase in men, 4.9% decrease in women, 9.1% decrease in patients < 65 years old and 9.1% increase in patients ≥ 65 years old.”

