# Supplementary material for: Changes in Distribution of Dry Eye Disease by the New 2016 Diagnostic Criteria from the Asia Dry Eye Society
Source: Sci Rep. 2018 Jan 30;8:1918. doi: 10.1038/s41598-018-19775-3 (PMC5789837; doi:10.1038/s41598-018-19775-3)

Title: Changes in Distribution of Dry Eye Disease by the New 2016 Diagnostic Criteria from the Asia Dry Eye Society.

Authors: *Takenori Inomata[1,2,*] MD, PhD, MBA, Tina Shiang[3] MD, Masao Iwagami[4] MD, MPH, Msc, Fumika Sakemi[1] MD, Keiichi Fujimoto[1] MD, Yuichi Okumura[1] MD, Mizu Ohno[1] MD, and Akira Murakami[1] MD, PhD.*

Supplementary Information

Supplemental Table 1. The changes in the distribution of DED subgroups in (A) men, (B) women, (C) patients <65 years old, (D) patients >65 years old and Sjogren's syndrome between the 2006 and 2016 criteria. DED; dry eye disease.

A.

Men	2006 Criteria				
2016 Criteria	Classification	Definite DED	Probable DED	Non-DED	Total
	Definite DED	8 (88.9)	17 (89.5)	0 (0)	25 (44.6)
	Non-DED	1 (11.1)	2 (10.5)	28 (100)	31 (55.4)
	Total	9 (100)	19 (100)	28 (100)	56 (100)

B.

Women	2006 Criteria				
2016 Criteria	Classification	Definite DED	Probable DED	Non-DED	Total
	Definite DED	42 (100)	46 (78.0)	0 (0)	88 (65.2)
	Non-DED	0 (0)	13 (22.0)	34 (100)	47 (34.8)
	Total	42 (100)	59 (100)	34 (100)	135 (100)

C.

<65	2006 Criteria				
2016 Criteria	Classification	Definite DED	Probable DED	Non-DED	Total
	Definite DED	30 (96.8)	26 (86.7)	0 (0)	56 (69.1)
	Non-DED	1 (3.2)	4 (13.3)	20 (100)	25 (30.9)
	Total	31 (100)	30 (100)	20 (100)	81 (100)

D.

≥65	2006 Criteria				
2016 Criteria	Classification	Definite DED	Probable DED	Non-DED	Total
	Definite DED	20 (100)	37 (77.1)	0 (0)	57 (51.8)
	Non-DED	0 (0)	11 (22.9)	42 (100)	53 (48.2)
	Total	20 (100)	48 (100)	42 (100)	110 (100)

E.

Sjogren's syndrome	2006 Criteria				
2016 Criteria	Classification	Definite DED	Probable DED	Non-DED	Total
	Definite DED	46 (100)	8 (72.7)	0 (0)	54 (91.5)
	Non-DED	0 (0)	3 (27.3)	2 (100)	5 (8.5)
	Total	46 (100)	11 (100)	2 (100)	59 (100)

Supplemental Fig 1. Changes in the scatter plot of Schirmer test I values and TBUT among four subtypes of patients with DED among Sjogren's syndrome per the 2006 and 2016 criteria. Figures show the scatter plot of TBUT (X axis, second) and Schirmer test (Y axis, mm). TBUT; tear break-up time.

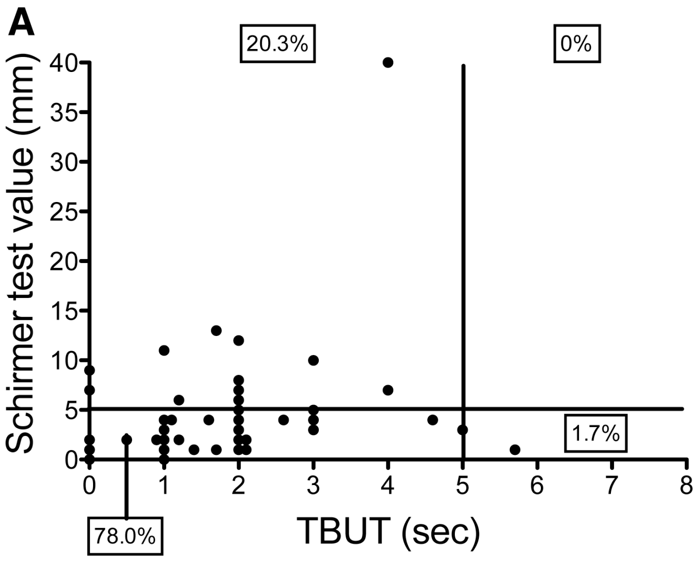

Supplement: Supplementary file 1 — Supplementary information [file 41598_2018_19775_MOESM1_ESM.pdf]
